# Plasma nevirapine concentrations predict virological and adherence failure in Kenyan HIV-1 infected patients with extensive antiretroviral treatment exposure

**DOI:** 10.1371/journal.pone.0172960

**Published:** 2017-02-24

**Authors:** Maureen J. Kimulwo, Javan Okendo, Rashid A. Aman, Bernhards R. Ogutu, Gilbert O. Kokwaro, Dorothy J. Ochieng, Anne W. T. Muigai, Florence A. Oloo, Washingtone Ochieng

**Affiliations:** 1 Center for Research in Therapeutic Sciences, Strathmore University, Nairobi, Kenya; 2 ITROMID, Jomo Kenyatta University of Science and Technology, Nairobi, Kenya; 3 Institute of Healthcare Management, Strathmore University, Nairobi, Kenya; 4 African Centre for Clinical Trials, Nairobi, Kenya; 5 Kenya Medical Research Institute, Nairobi, Kenya; 6 School of Pharmacy, MCPHS University, Worcester, Massachusetts, United States of America; 7 Department of Chemical Science and Technology, Technical University of Kenya, Nairobi, Kenya; 8 Immunology and Infectious Diseases Department, Harvard School of Public Health, Boston, Massachusetts, United States of America; University of Maryland School of Medicine, UNITED STATES

## Abstract

Treatment failure is a key challenge in the management of HIV-1 infection. We conducted a mixed-model survey of plasma nevirapine (NVP) concentrations (cNVP) and viral load in order to examine associations with treatment and adherence outcomes among Kenyan patients on prolonged antiretroviral therapy (ART). Blood plasma was collected at 1, 4 and 24 hours post-ART dosing from 58 subjects receiving NVP-containing ART and used to determine cNVP and viral load (VL). Median duration of treatment was 42 (range, 12–156) months, and 25 (43.1%) of the patients had virologic failure (VF). cNVP was significantly lower for VF than non- VF at 1hr (mean, 2,111ng/ml vs. 3,432ng/ml, p = 0.003) and at 4hr (mean 1,625ng/ml vs. 3,999ng/ml, p = 0.001) but not at 24hr post-ART dosing. Up to 53.4%, 24.1% and 22.4% of the subjects had good, fair and poor adherence respectively. cNVP levels peaked and were > = 3μg.ml at 4 hours in a majority of patients with good adherence and those without VF. Using a threshold of 3μg/ml for optimal therapeutic nevirapine level, 74% (43/58), 65.5% (38/58) and 86% (50/58) of all patients had sub-therapeutic cNVP at 1, 4 and 24 hours respectively. cNVP at 4 hours was associated with adherence (p = 0.05) and virologic VF (p = 0.002) in a chi-square test. These mean cNVP levels differed significantly in non-parametric tests between adherence categories at 1hr (p = 0.005) and 4hrs (p = 0.01) and between ART regimen categories at 1hr (p = 0.004) and 4hrs (p<0.0001). Moreover, cNVP levels correlated inversely with VL (p< = 0.006) and positively with adherence behavior. In multivariate tests, increased early peak NVP (cNVP4) was independently predictive of lower VL (p = 0.002), while delayed high NVP peak (cNVP24) was consistent with increased VL (p = 0.033). These data strongly assert the need to integrate plasma concentrations of NVP and that of other ART drugs into routine ART management of HIV-1 patients.

## Introduction

Scaling of antiretroviral treatment (ART) has significantly reduced deaths and morbidity for HIV patients. In resource-constrained settings, limited ART drug options still dampen long-term treatment success. Kenya’s standard ART regimen is still restricted to a limited combination options [1]. At the time of this study, ART qualifications and options were even more stringent [2], with regimen including one of Zidovudine (AZT), Stavudine (D4T) or Tenofovir (TDF) backbone administered in triple combination with Lamivudine (3TC) and either Nevirapine (NVP) or Efavirenz (EFV). NVP is a particularly widely prescribed component of highly active antiretroviral therapy (HAART) and is used alongside the other ART drugs to maximize viral suppression. Although a few studies have suggested that plasma NVP concentrations (cNVP) may affect virologic outcome [3–5], therapeutic drug level (TDL) measurements is excluded from HAART management of patients in many countries, including Kenya. We recently showed in a larger Kenyan HIV population that up to 81% of the patients were NVP experienced, with a significant proportion as high as 35% failing treatment and exhibiting poor adherence [6]. Another study of Western Africa and Asian populations showed highly heterogenous virologic failure across different population sites [7], an observation that may be associated with variable treatment and adherence behaviors. Factors like prescribing practice, ART stock outs, promptness of refill pickup, are known to significantly affect treatment outcomes [8]. Moreover in Kenya like many parts of the world, patient behavior affects adherence reporting and measurement [9]. The success of any ART requires optimal drug exposure on one hand [10], while on the other hand; extensive use of, or suboptimal exposure to NVP presents various risks to the success of treatment [5, 11, 12]. Besides our studies, others have also shown that patient behavior carries even greater sway on the veracity of adherence measured on the basis of self-report and pill counts [13–17]. It is clear from the preponderance of evidence that multiple factors converge to influence treatment outcomes. While therapeutic concentrations of anti HIV drugs positively predict virologic failure (VF) [18], few if any of these studies integrate TDL or pharmacologic measurements into adherence and VF monitoring. Even in patients reporting good adherence, some demonstrate subtherapeutic concentrations of drug that confounds interpretation and application of adherence data, often largely due to high interpatient variability [19, 20]. Such variability can be partly due to differences in host genetics and drug metabolism [21], thus supporting the need for TDL monitoring to bolster clinical management of HIV [22]. Specifically, long elimination half-life of NVP and its low genetic barrier to resistance may dampen its long-term therapeutic efficacy [11, 23]. Therefore, it is crucial that patients achieve and maintain efficacious plasma drug concentrations to circumvent viral breakthrough. We argued that since adherence is crucial to maintaining adequate levels of blood drug levels in order to achieve a good ART response, routine TDL monitoring of circulating blood NVP could provide a more reliable proxy for adequate assessment of compliance to medication that positively affects treatment outcome. Since TDL monitoring is not available in most resource-constrained settings outside of limited research contexts [24–26], we conducted this study in order to develop a comprehensive understanding of how cNVP relates with adherence and treatment outcomes. Specifically, we aimed to assess if cNVP would accurately predict virologic treatment outcome and if that predictive value was a proxy measure of adherence in these long-term HAART patients receiving nevirapine as part of a 3-drug regimen.

## Materials and methods

### Subjects and sampling

This was a mixed method study involving both quantitative and qualitative data collection. We initially enrolled 1600 subjects across multiple study sites in Kenya into 4 interdependent sub-studies. The first 3 components of that study focused on describing the correlates of adherence and treatment outcome and genotypic variability and have been published [6, 27, 28]. About 500 of the larger population were in active ART register at the two study sites also used for NVP drug level studies. Given lack of national data on treatment failure rates from which sampling guidance could be based, and due to limited resources we developed a conservative hypothesis that 60% of the 500 patients would provide a correct ART adherence response, and that because of inherent stigma, this disclosure would have a wide margin of error of 10%. We thus included 58 patients from these two sites, to give us a 90% power to detect 10% error in self-reported adherence (proxy for treatment failure). These patients are not part of data already reported and were aged 12 to 73 years. Sampling for plasma nevirapine concentrations (cNVP) was done at three time-points over a period of 24 hours, while that for viral load (VL) was achieved prospectively over two-time points that were 12 (median) months apart and used to determine virologic failure. With 3 pharmacokinetic sampling points, this strategy would allow us to detect 5% error margin on virologic failure attributable to adherence reports, with 90% confidence.

### ART regimen and data collection

All HIV-infected adult subjects who were receiving NVP as one of the 3-drug ART regimen and returning to the clinic for refills were included on a convenience sampling basis. Fifty-five of the 58 subjects enrolled in this study component were on one of three recommended first-line treatment [29]: nineteen (32.8%) on 300mg zidovudine (AZT) + 150mg lamivudine (3TC) + 200mg NVP; sixteen (27.6%) on stavudine (D4T, 40mg) + 3TC + NVP; and 20 (34.4%) received tenofovir (TDF, 300mg) + 3TC +NVP. All these regimens were administered twice daily. Three subjects we on non-standard ART regimen; one each receiving TDF+NVP or 3TC+NVP dual regimen and one receiving a twice-daily single NVP regimen. These three have been excluded from data analyses. Study personnel in conjunction with attending clinicians ensured patients on the study adhered to the complete twice-daily dose within the 24hr window of the pharmacokinetic analysis. Structured questionnaires were administered to collect all socio-demographic and clinical data including gender, age, adherence, regimen and ART duration, and participation in community peer support (CPS) programs. The details of CPS programs and data collection methodologies can be found in our earlier publication [6]. Five milliliters EDTA blood were collected from each subject at 1, 4 and 24 hours post dosing with the regularly assigned ART regimen that also contained NVP. Plasma and PBMCs were separated from the rest of the blood by Ficoll gradient centrifugation within 1 hour, and frozen immediately in replicate vials at -80°C until needed.

### HIV-1 viral load assays

VL assays were conducted withing 2 months of sample collection in duplicate. Analyses were done at the Early Infant Diagnosis Laboratory at the Kenya Medical Research Institute in Nairobi using Abbott RealTime HIV-1 assay system following manufacturer’s protocol (Abbott, Abbot Park, IL) and using an Abbott *m*2000*sp* platform as we described earlier. In brief, internal control RNA was added to 200μL of plasma and the sample loaded onto the *m*2000*sp* instrument for RNA extraction. The limits of detection for the *m*2000*sp* ranged between 40 and 10,000,000 HIV-1 RNA copies.

### Adherence and treatment failure definition

The World Health Organization (WHO) provides three criteria for treatment failure definition that includes clinical failure, immunological failure, and virological failure [30]. We used the virological criterion for its comparable sensitivity. Virologic failure (VF) was defined based on two consecutive VL measurements (VL1 and VL2- 12 months apart), and virologic success as the absence of VF. Subjects were considered to have failed treatment if they had both VL1 and VL2 that were persistently above 1000 copies/ml or if their VL1 was below 1000 copies/ml and VL2 above 1000 copies/ml. The two study facilities also offered optional adherence and compliance programs that relied on community peer support (CPS) mechanisms. The CPS groups comprise HIV+ peer ‘counselors’, who provide counseling and adherence support, including patient home visits and focused group discussions relevant to treatment compliance. The CPS councilors also verify pill count at refill and pill burden. Participation in these groups is voluntary, and patients were asked if they were actively, partly, or never involved CPS. Adherence assessment was based on residual pill count and on self-report, focusing on dose-compliance during the 30 days preceding the latest refill. The number of dose pills at refill was counted and reconciled against the dose counts dispensed at last refill. Additional pill count data was extracted from patient cards for the four months preceding the study period. Non-adherence was determined as the percentage of overdue dose at refill, averaged over a four-month period and used to assign adherence as good (< = 5% dose skipped), fair (6–15% dose skipped) or poor (>15% dose skipped) ART Adherence was assessed based on self-report and on pill burden, and defined as good (< = 5% dose skipped), fair (6–15% dose skipped) or poor (>15% dose skipped) as we have described recently [6].

### Determination of plasma NVP concentrations (cNVP)

We developed an in-house liquid-liquid extraction protocol for NVP based on modification of a previous method [31]. Frozen plasma were analyzed within 3 months of blood collection. Drug level analyses were performed using an Agilent 1260 High-Performance Liquid Chromatography (HPLC, 1260) with dual-wavelength UV—VIS spectrophotometric detector (Agilent, Santa Clara, CA), using a validated procedure developed in our laboratory. For any patient, all the three PK time points (1, 4 and 24 hours post dosing) were analyzed in same batch. NVP was extracted from plasma and analyzed in duplicate. The mobile phase was a 15mM potassium phosphate buffer/acetonitrile solution (70/30% v/v) with pH adjusted to 3 using orthophosphoric acid. Chromatographic conditions consisted of a 1.5ml/minute elution flow rate on a 5μm 150x4.6mm C18 column at 254nm wavelength. A total of 20 μl samples were injected into the column, with the entire run time lasting 5 minutes per sample. All standards were derived from 200mg NVP tablets (Boehringer Ingelheim, Ridgefield, CT, U.S), and provided by the National Drug Quality Control Laboratory (NDQCL) in Nairobi. The NDQCL regularly tests all HIV drugs destined for use in the Kenyan population, for bioequivalence and safety standards and the NVP tablets obtained from the NDQCL for use as standards had met these criteria. A single tablet was weighed, crushed and re-weighed then reconstituted in HPLC grade acetonitrile to make a stock standard solution (SS, 10mg/ml). The SS was used to make a diluted working standard (WS, 0.1mg/ml). Calibration curves were prepared daily in blank healthy human plasma and were linear over the range 0–10μg/ml (0, 1, 2, 4, 6, 8, 10 μg/ml). Three quality control standards were similarly prepared at 1.5, 5.5 and 8.5 μg/ml concentrations.

### Statistical analysis and data quality

All patients in this study were actively receiving antiretroviral therapy (ART) at the designated clinics/facilities and their sampling was on convenience basis to include any consenting subjects meeting recruitment criteria outlined elsewhere [6]. Chromatographic readouts in units of area under the curve (AUC) of NVP were converted into standard concentrations units (ng/ml) using machine software integral to the HPLC-MS system. To ensure integrity of the data, the calibration curve used to generate concentrations of unknown samples were prepared daily alongside processed samples. Additionally, all stock solutions and working solutions were prepared in triplicates and stored under -20°C to be used within a month. Finally, validation of the HPLC protocol was performed prior to sample analysis to ensure the repeatability of the results, accuracy of the extraction process and recovery. Patient demographic and treatment characteristics were analyzed using descriptive statistics. Outcome (dependent) variables included VL and NVP plasma concentrations (cNVP) at 1, 4 and 24 hours post-dosing (cNVP1, cNVP4, and cNVP24). Independent (categorical) variables were age, sex, virologic failure outcome (VF), ART regimen, adherence and community peer support (CPS) groups. We used both non-parametric Mann-Whitney’s U-test and Kruskal-Wallis test to test the null hypothesis that the distribution of outcome variables such as VL or cNVP, was similar respectively across categories of 2 independent variables (e.g. virologic failure and virologic success) or categories of K (>2) independent variables (such as good, fair or poor adherence). Analysis of variance (ANOVA) was used to assess the difference in means of outcome variables between sets of independent variables. Pearson correlation analysis was used to assess the associations between outcome variables (e.g cNVP and VL). Chi-square tests were used to determine associations between two independent variables. Multivariate analysis of variance (MANOVA) tests were conducted within the general linear model to assess independent associations between variables. All the four MANOVA associated statistics were considered, and where F-values differed, Pillai’s test is reported due to its relative robustness. All analyses were performed using SPSS version 22. Results were significant if p-value was less than 0.05.

### Ethical consideration

This study and the consent procedure was approved by the Scientific and Ethical Review Unit (SERU) of the Kenya Medical Research Institute. Subjects 18 years or older were required to sign a written voluntary informed consent while a written voluntary informed assent of minors (<18 years) was provided on their behalf by legal guardians/parents and approved SERU.

## Results

### Baseline demographic and treatment characteristics of patients

There were twice as many females as males, with the overall median duration of treatment for all 58 subjects of 42 months (range 12–156). In total, 19 (32.8%) subjects received AZT+3TC+NVP, 16 (27.6%) received D4T+3TC+NVP and 20 (34.5%) received TDF+3TC+NVP regimens. Two subjects (3.4%) were on NVP+TDF and 3TC+NVP regimens and one (1.7%) on NVP alone. These 3 subjects not receiving the optimal triple ART regimen also had the highest baseline viral load (VL1) of 3.51 log_10_ HIV RNA copies compared to the other regimen groups. No reason was on record for this apparent non-standard therapy. Another 53.4%, 24.1% and 39.7% had good, fair poor adherence respectively. Twenty-three (39.7%) subjects were actively involved in community peer-to-peer support (CPS) program, 17 (29.3%) only occasionally participated in CPS while 18 (31.0%) never participated in CPS. The mean baseline VL (VL1) was log_10_ 3.33 copies, and was comparable for males (log_10_ 3.40 copies) and females (log_10_ 3.30 copies). To avoid any confounders to VF that might arise from suboptimal therapy, the three subjects have been excluded from statistical data analyses.

### Adherence and Virologic Failure (VF)

VF was defined using 2 successive viral load (VL) measurements as in methods. The median duration between VL1 and VL2 was 12 months for this study subset. By this definition, 25 (43.1%) of the patients failed treatment. Comparing VF outcome across ART regimen and adherence, patients receiving D4T backbone had significantly higher VL (mean VL2 4.11 log_10_ copies) than patients on other treatment arms (p = 0.006). Defining adherence as good, fair or poor, 31 patients (53.4%) had good adherence, 14 (24.1%) had fair adherence while 13 (22.4%) had poor adherence. Adherence was strongly associated with VF outcome in a chi-square test (p-value <0.001). Subjects with good adherence also had the lowest VL (2.4log_10_ copies/ml) compared to those with fair (3.31 copies/ml) or poor (4.8 copies/ml) adherence. These VLs were significantly different as detailed in Table 1 (p<0.001).

**Table 1:** Virologic treatment response of various categories of patients.

		VL1; VL2_‡_, N, (%)		
		VF	VS	Total = 100%
Gender	Males	4.35; 4.44,6 (31.6)	2.96; 2.59, 13 (68.4)	3.40; 3.17,19
	Females	3.43; 4.15, 17 (47.2)	3.15; 2.20, 19 (52.8)	3.28; 3.12, 36
	p value_¶_- VL1| VL2			0.675 | 0.898
Age	≤25	0, 0 (0)	3.71; 2.42,3 (100)	3.71; 2.42, 3
	26–45	3.65; 4.32,15 (46.9)	3.15; 2.59,17 (53.1)	3.39; 3.41, 32
	>45	3.71; 4.05,8 (40)	2.8; 2.02,12 (60)	3.16; 2.83, 20
	p value_¶_- VL1| VL2			0.53| 0.219
Regimen_†_	AZT	3.89; 3.75,7 (36.8)	3.22; 2.08,12 (63.2)	3.47; 2.69, 19
	D4T	3.77; 4.87,10 (62.5)	2.98; 2.87,6 (37.5)	3.47; 4.12, 16
	TDF	3.26; 3.72,6 (30)	2.98; 2.39,14 (70)	3.06; 2.79, 20
	p value_¶_- VL1| VL2			0.284 | 0.002
Adherence	Good	3.26; 2.97,5 (17.9)	3.0; 2.14, 23 (82.1)	3.04; 2.29, 28
	Fair	3.82; 4.37, 6 (42.9)	3.3; 2.51,8 (57.1)	3.52; 3.31, 14
	Poor	3.77; 4.68, 12 (92.3)	3.07; 6.27, 1 (7.7)	3.72; 4.80, 13
	p value_¶_- VL1| VL2			0.05 | <0.001
CPS	Active (++)	3.30; 3.30, 2 (9.0)	2.86; 2.25, 20 (91.0)	2.9; 2.34, 22
	Occasional (+)	3.28; 3.77, 7 (46.7)	3.49; 2.45, 8 (53.3)	3.39; 3.07, 15
	None (-)	3.92; 4.59, 14 (77.8)	3.29; 2.76, 4 (22.2)	3.78; 4.18, 18
	p value_¶_- VL1| VL2			0.008 | <0.001

*Others is used to describe patients who were on NVP+TDF, 3TC+NVP or NVP;

^†^, Regimen backbone includes 3TC+NVP;

^‡^, VL2 represents log_10_ copies/ml viral load taken at the second study time-point after VL1;

^¶^, ANOVA p-value for comparison of mean VL2 between independent variables.

We next assessed the associations between participation in CPS and VF. A total of 22 subjects (40%) were active in CPS (CPS++), 15 (27.2%) were partly involved (CPS+) and 18 (32.7%) were never involved (CPS-). CPS activity was significantly associated with VF outcome (χ^2^ p<0.001). Specifically, 90.9% CPS++ patients experienced virologic success (non-VF) compared to just 22.2% of CPS- patients experiencing virologic success. Similarly, CPS++ subjects had the lowest mean VL (2.40 log 10) compared to CPS+ (3.22 log10 copies/ml) or CPS- (4.18 log10 copies/ml) subjects, and these differences were significant in an ANOVA test (p<0.001). Moreover, adherence and CPS were positively correlated in a chi-square test (p<0.001), with more patients in CPS++ showing good adherence and more CPS- patients showing poor adherence. Age and gender were not significantly associated with VF outcome.

### Trajectory of plasma NVP concentrations

**Fig 1:**
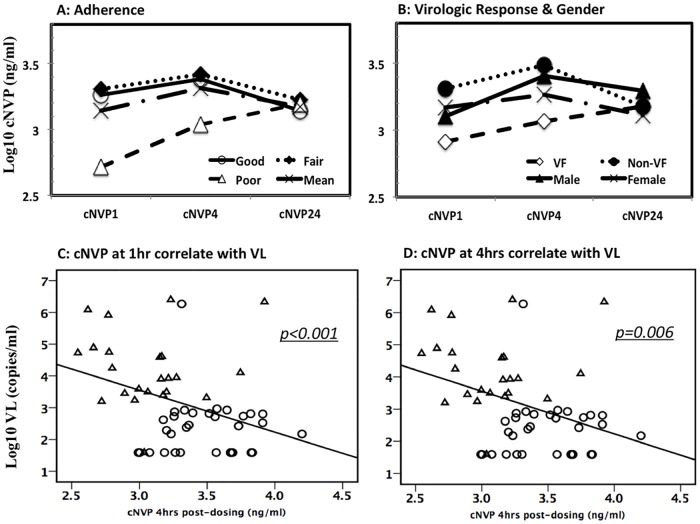
Trajectory plasma nevirapine concentrations and association with viral load. Plasma nevirapine concentration (cNVP) is compared for various groups over 24 hour period according to adherence (A:- open circle, good adherence; closed diamonds, fair adherence; open triangle, poor adherence; solid line, mean), and according to virologic response or gender (B:- open diamond, virologic failure; closed circle, virologic success or non-virologic failure; closed triangle, male; crosses, female). Patients with good and fair adherence and those with virologic success (non-virologic failure) had peak cNVP at 4 hours while cNVP for virologic failure patients started low at 1hr and peaked later at 24hrs. Significant inverse correlations are observed between same day viral load with cNVP at 1 hour (C) and at 4 hours (D). Circles, virologic success; triangles, virologic failure.

Existing data suggest that NVP concentrations of 3.0 μg/ml may be an optimal threshold on the basis of steady-state trough concentration reached in the pharmacokinetic curve for NVP following a 200mg twice daily dose [5, 10, 26, 32, 33]. Here, the trajectory of cNVP showed a steady rise from low levels at 1 hour, and peaking at 4 hours before tapering off 24 hours after dosing. On average, and in a significant proportion of patients experiencing good and fair adherence, as well as those with virologic success, cNVP peaked at 4 hours post ART dosing. These peak cNVP levels were either equal to or above the threshold of 3μg/ml concentration. This pattern held largely true when comparing cNVP by adherence (Fig 1A), gender or VF outcome, except for subjects with poor adherence and those with virologic failure who experienced delayed peak cNVP. Patients experiencing virologic failure and those with poor adherence had lower cNVP that rose slowly but steadily before peaking at 24 hours. Those experiencing virologic success (non- virologic failure) had cNVP starting about 5-fold higher than virologic failure patients, but peaking early at 4hrs before tapering off at 24 hours (Fig 1B). Overall, cNVP at 24 hours was comparable regardless of adherence, VF status or gender. All virologic failure patients, as well as those with poor adherence appeared unable to sustain cNVP above 3ug/ml within the first 4 hours of ART dosing.

### Plasma NVP concentrations are associated with adherence outcome

We next examined the relationship of cNVP with adherence outcome. Mean cNVP was at least two-fold higher in patients with good (3,392–3344 ng/ml) and fair (3,368–3,433 ng/ml) adherence compared to those with poor adherence (1,278–1,638 ng/ml) (Table 2). Both cNVP1 and cNVP4 were significantly less for patients with poor than those with fair or good adherence (Anova p< = 0.009). Non-parametric tests were used to test the null hypothesis that the distribution of cNVP was similar across adherence groups. These between-groups comparison revealed that cNVP1 (p = 0.005) and cNVP4 (p = 0.014) were significantly different across adherence groups. At the time when majority of patients experienced peak cNVP levels at 4hrs post-ART dosing, achieving cNVP at or above 3μg/ml coincided with good adherence and virologic success. Using 3μg/ml as threshold for optimal or therapeutic NVP drug levels (TDL), we grouped patients into either sub-therapeutic (<3μg/ml cNVP) or therapeutic (> = 3μg/ml cNVP) categories and determined the association of TDL with adherence. By this analysis, the proportion of patients with poor adherence (n = 13) and having sub-therapeutic cNVP (12/13, 92.3%) was higher than those achieving therapeutic cNVP under the same poor adherence category (1/13, 7.7%, p = 0.05). This relationship between TDL and adherence was significant at peak cNVP at 4 hours but not at 1 or 24 hours post-ART dosing. No significant association was found between TDL and age group or gender, or between TDL24 and any grouping variables.

**Table 2:** Peak nevirapine concentration is consistent with better adherence and viral load suppression.

Grouping variable		cNVP in ng/ml, post-dosing at		
		1 hour	4 hours	24 hours
Adherence	Good	3391.72	3343.86	1872.36
	Fair	3368.36	3433.21	2356.75
	Poor	1277.75	1637.68	2356.75
	p value_¶_	0.002	0.009	0.788
CPS activity	Active (++)	3983.01	3374.63	1234.62
	Occasional (+)	2163.47	2884.92	3028.17
	None (-)	2147.67	2526.00	6168.36
	p value_¶_	0.078	0.384	0.123
Virologic response	ART failure (25)	2164.90	1660.47	5056.10
	ART responsive (33)	3404.48	3899.75	2103.89
	p value_¶_	0.004	<0.001	0.972
Gender	Males	2058.38	3259.43	6319.89
	Females	3322.97	2807.04	1764.91
	p value_¶_	0.657	0.192	0.108
Age group (years)	≤25	2604.80	3828.60	5315.15
	26–45	2424.83	3383.73	4431.59
	>45	3666.36	2160.87	1292.83
	p value_¶_	0.642	0.495	0.107
Regimen arm_‡_	AZT	1496.07	2878.39	5985.37
	D4T	2280.63	2042.53	2075.74
	TDF	4691.05	3780.65	1834.04
	p value_¶_	0.042	0.56	0.910

^¶^Anova p value compares mean log10 plasma nevirapine concentrations (cNVP) between grouping variables. CPS, community peer-to-peer support.

^‡^Regimen arm includes 3TC+NVP in the ART backbone

### Associations of cNVP with virologic and treatment outcome

We also examined the relationship of cNVP with treatment outcome, and these were significantly lower for cNVP1 (p = 0.004) and cNVP4 (p<0.001) but not cNVP24 in VF patients than in patients not experiencing (Table 2) VF. Compared between regimen arm, patients receiving AZT and D4T had 2 to 3-fold less cNVP than those on TDF backbone, though these differences were only significant at 1 hour (p = 0.042). We applied the therapeutic drug level (TDL) threshold in which cNVP <3μg/ml represented sub-TDL and > = 3μg/ml represented optimal TDL at any of the three pharmacokinetic time points (TDL1, TDL4 & TDL24). Majority of the patients had cNVP below 3μg/ml at 1 hour (41/55, 74.5%), at 4 hours (36/55, 65.5%) and at 24 hours (48/55, 87.3%). Applying this threshold to test for independent variable associations, TDL4 (χ^2^ p = 0.004) but not TDL1 or TDL24 was significantly associated with virologic failure. Of subjects experiencing VF, significantly more had sub-therapeutic NVP levels at the 4-hour peak (82.6%, n = 19/23) compared to just 9.4% (n = 3/32) who achieved optimal TDL yet still failing treatment. Mean VL was significantly lower for subjects who had cNVP4 at or above this threshold (n = 19, mean log_10_ 2.64) compared to those with cNVP4 below the threshold (n = 36, mean log_10_ 3.41 3μg/ml, p = 0.049). Similar analyses conducted for cNVP taken at 1hr or at 24hrs post-dosing were not significant except where otherwise mentioned. We also fitted cNVP1, cNVP4 and cNVP24 alternately into a bivariate correlation model with VL at the time of cNVP determination as the other scale variable. VL was significantly and inversely associated with cNVP1 (p<0.001, Fig 1C) and with cNVP4 (p = 0.006, Fig 1D) but not with cNVP24.

### Multivariate associations

Multivariate tests were conducted within the general linear model to assess independent associations between variables, and at least to validate the associations seen using chi square tests. Adherence and VF outcome were fitted into the step-wise model as independent variable while cNVP1, cNVP4, cNVP24, VL1 and VL2 were fitted as outcome variables. Both adherence (p = 0.004) and VF (p = 0.031) were independently and strongly associated with these outcomes (Table 3). Between subject effects revealed cNVP at 4 hours (p = 0.001) and VL2 measured at the time of cNVP sampling (p = 0.009) to both be strongly predictive of whether or not the patients were defined as VF, with patients likely to fail treatment if they either had low cNVP or high VL. However, only VL (p< = 0.001) but not cNVP was associated with adherence in these tests. Conversely when therapeutic NVP level was fitted into the model as independent variable (described as above or below 3ul optimal threshold) and viral load as outcome, patients who had delayed peak NVP levels (i.e cNVP24), also had significantly high VL (p = 0.033), although this relationship did not predict whether or not those patients were defined as virologic failures.

**Table 3:** Peak cNVP predict virologic response as much as does VL in a multivariate analysis.

**Multivariate Tests**^**a**^					
***Predictor***	Value	F	df	Error df	*p*-value
Adherence	.672	3.742	10.000	74.000	< .001
VF	.357	3.992	5.000	36.000	.006
Adherence * VF	.382	1.747	10.000	74.000	.086
**Tests of Between-Subjects Effects**					
	Type III Sum of Squares	df	Mean Square	F	*p*-value
***Factors associated with virologic failure (VF)***					
Viral load 1 (VL1)	1.213	1	1.213	1.522	.224
Viral load 2 (VL2‡)	3.071	1	3.071	6.366	.016
cNVP4^†^	1.221	1	1.221	11.169	.002
***Factor associated with adherence***					
VL1	.907	2	.453	.569	.571
VL2	14.849	2	7.424	15.389	< .001

Multivariate tests are shown with VL and cNVP as scale variables. VF and Adherence are factors. Effect of the predictors on outcome is shown at the bottom of the table.

^†^ Only cNVP at 4 hours predicted VL and VF.

^‡^VL2 is median 12 months from VL1, and at same time as cNVP data point. Tests with therapeutic NVP level as independent variable is not included in this table, but described in text.

## Discussions

We conducted this study to investigate associations of cNVP with treatment and adherence outcome among Kenyans receiving NVP as part of a long-term ART regimen. More than half (53.4%) of these patients had good adherence. Patients with virologic success achieved significantly high cNVP within one hour of dosing compared to delayed cNVP peak for those with virologic failure. Conversely, patients failing treatment showed poor adherence and significantly reduced cNVP. Moreover, delayed but not early peak cNVP was independently predictive of virologic failure while early peak cNVP was predictive of virologic success in multivariate analyses. We recently reported high virologic failure in a larger population of Kenyans who received HAART for between 12 and 228 months [6]. In the present study, patients were specifically enrolled as part of that larger study population to include only those receiving NVP in their ART backbone, but which were not included in the previous report.

NVP is extensively used in Kenya as part of first-line therapy, and VL monitoring for ART management is accessible only to a limited extent. Moreover, neither drug level nor toxicity monitoring is included in clinical management of HIV patients. The median duration between VL1 and VL2 for this study subset was 12 months, which was in line with the average duration of VL measurement intervals for clinical ART management in Kenya. This study however,. expands virologic failure ssessment to include therapeutic drug level (TDL) monitoring, and the longer duration between VL1 and VL2 would otherwise not change the virologic response status determination, although failing patients may not benefit from timely clinical decisions. We show that adherence significantly affects virologic outcomes and plasma cNVP: good adherence was associated significantly with suppressed VL and higher cNVP while poor adherence was associated with significantly high VL and lower (sub-therapeutic) cNVP levels. Moreover, TDL was predictive of virologic and adherence outcomes; attainment of early peak cNVP (4 hours post dosing) was significantly consistent with lower viral load and predictive of virologic success while patients achieving delayed peak cNVP (24 hours) were likely to fail treatment in multivariate tests. In a study conducted in Malawi to examine factors that affect treatment outcomes in patients receiving ART, up to 47% of the subjects were non-adherent to treatment [34]. That study also showed adherence to be significantly associated with subtherapeutic NVP plasma levels and with virologic failure. Adherence in our study was measured by a combination of pill burden and self-report, with a total of 77.5% being labelled as either good or fair adherents. Although our study was not powered to define the effective therapeutic NVP levels, mean cNVP was at least two-fold higher for patients with either good or fair adherence compared to poor adherence. cNVP did not however, distinguish between fair and good adherence, likely due to the blurry response and disclosure boundaries of these two categories. Importantly, a significant majority of patients who experienced virologic success and those who had good adherence also achieved cNVP of at least 3μg/ml four hours after initial ART dosing and significantly suppressed their plasma viral load. These data provide evidence to support the recommendation that therapeutic NVP level monitoring should be included as part of ART management, especially in settings where this drug is extensively used in suppressive HAART [35, 36].

Accumulating evidence show that community support networks (CPS) enhances social relationships that demystify HIV-associated stigma [37, 38]. We previously showed that participation in CPS networks significantly improved adherence and treatment outcome [6]. In this current subset of patients, those actively involved in CPS tended to reach peak cNVP early at 4 hours post-dosing, and these cNVP also were substantially higher than seen in patients not actively involved in CPS. We believe that CPS affected cNVP outcome by influencing adherence, a secondary relationship observed in our earlier larger patient population. Regimen was an important factor affecting virologic response, with more (62.7%) subjects receiving the D4T backbone also significantly failing treatment. The use of D4T has been banned in many industrialized countries because of its toxicities [39], and we have recommended fast-tracking of its phase-out in Kenya [6]. A comparison of regimen with cNVP revealed a tendency towards association of both the regimen containing AZT and D4T, with lower cNVP. At the time of this study, AZT was the first drug of choice for majority of patients initiating or changing regimen, while D4T was widely used due to its lower cost and increased availability. We suspect that the widespread use of D4T despite known underlying toxicities, may have contributed to poor ART compliance and by extension cNVP bioavailability in patients receiving a D4T backbone. Furthermore, statistical tests suggested that cNVP1 and cNVP4 significantly differed between adherence and virologic response categories, bolstering an argument for a stronger relationship between cNVP and treatment outcome.

ART management in Kenya at the time of the study followed existing national guidelines that included periodic monitoring of VL and CD4 levels [29], but did not include TDL monitoring as is often the case in most developed country settings [40]. This study provides the first evidence in the local context, that TDL can be readily incorporated into HIV management to monitor treatment outcomes and inform ART decisions. Patients who controlled viremia and those who had good adherence achieved early peak plasma cNVP than those with virologic failure or poor adherence. We believe that the ability of patients to achieve early rather than long-term cumulative or delayed therapeutic plasma drug concentrations is necessary to sustain viral suppression and treatment success in these patients receiving nevirapine twice daily as part of triple ART regimen. It is reasonable to suggest on the basis of these data, that NVP and other ART drug concentrations in blood be monitored in a structured clinical setting, particularly in patients not suppressing VL within the first 6–12 months of HAART initiation. While cost and intensity of such a protocol may seem prohibitive in most resource limited setting, patients can be selected that meet virologic or clinical criteria approaching or meeting treatment failure. Such patients can benefit from informed decision on drug selections to improve therapeutic efficacy. Our results should be interpreted within the limits of the study design and context. The study was not designed for intensive pharmacokinetics (PK) analyses, which is otherwise not practical in a clinical setting as a routine TDL monitoring practice. Under intensive PK studies, the VMax and trough levels for cNVP might deviate from what we have observed, but these are unlikely to affect overall conclusions. Moreover, the determination of circulating NVP levels using approaches applied in this study does not discriminate between sub-therapeutic drug concentrations due to poor adherence or differences in liver metabolism. In addition, these data should not be extrapolated to other ART drugs, whose pharmacokinetics and responses are likely different. Notwithstanding, the findings remain appropriate for patients in similar setting receiving comparable regimens where resources and drug options are otherwise limited.
